# Body Representation in Children With Unilateral Cerebral Palsy

**DOI:** 10.3389/fpsyg.2019.00354

**Published:** 2019-02-19

**Authors:** Arturo Nuara, Pamela Papangelo, Pietro Avanzini, Maddalena Fabbri-Destro

**Affiliations:** ^1^Consiglio Nazionale delle Ricerche, Istituto di Neuroscienze, Parma, Italy; ^2^Dipartimento di Scienze Biomediche, Metaboliche e Neuroscienze, Università di Modena e Reggio Emilia, Modena, Italy

**Keywords:** childhood stroke, perinatal stroke survivors, self-body representation, self-portrait, body image, perinatal stroke

## Abstract

Drawings produced by children provide insights about their physical and psychological status. In children suffering from unilateral cerebral palsy (UCP), self-portraits constitute a unique opportunity to study whether and how their disease affects self-body representation. The aim of the present study is to evaluate self-body representation in UCP children, comparing it to the way they portray both healthy and hemiparetic peers. Ten UCP children were asked to perform 3 drawings: a self-portrait, a portrait of their best classmate, and finally a portrait of a hemiparetic peer who had joint them in a child-to-child rehabilitation protocol. As controls, 16 typically developing children were asked to perform a self-portrait, and their best-classmate portrait. The asymmetry index (AI), consisting of the difference between the upper limbs length expressed as percentage of their average, resulted greater in UCP than in controls’ self-portrait. More interestingly, UCP children portrayed themselves more asymmetrically relative to their classmates and hemiparetic peers. No difference in terms of AI was found between self- vs. classmate-portrait in the control group. This study provides evidence that UCP affects body self-representation, but not body-representation in general. In fact, the asymmetry in upper limb representation observed in children with UCP does not constitute a mere picturing of the hemiparesis, but rather reflects the experienced status of functioning, that is valid only for one’s own. The inclusion of portraits in pediatric neurorehabilitation programs might enable clinicians to collect additional evidence about the children self-perceived functioning, i.e., an information not easily obtainable in pediatric patients.

## Introduction

Children have been using drawings to express themselves since ancient times (Wittmann and Barber, 2013). The idea that spontaneous drawing of young children may reflect their physical, cognitive and affective status, led psychologists to exploit drawings as a useful tool for assessing child development, personality and emotional adaptation (Cooke, 1885; Goodenough, 1975; Matthews, 2003).

One of the most used methods to measure the level of development through drawing is the DAM test (Draw-a-man) (Goodenough, 1975), which is a projective test using portraits: drawing a person, a child “projects himself in all of the body meaning and attitudes that have come to be represented” (Machover, 1949).

The body image, regarded as the conscious representation of the body parts and their relative position, involves both the subject’s perceptual body experience with the body limits and conceptual understanding of the body in general (Gallagher, 2005). Parallel to the body image is the so-called body schema, i.e., the subconscious ideas about the shape and size of the body and the relationship of the parts of the body to each other. While both these aspects affect the human figure drawing, deficits specific for body schema or body image are very difficult to separate (de Vignemont, 2010). For this reason, several studies refer to overall disorders of body representation to collectively describe these concepts (Lampe et al., 2016).

Among neurological conditions, cerebral palsy (CP) is the one in which brain injury effects on body representation have been more extensively investigated by means of human figure drawing (see for example Lampe et al., 2016). Abercrombie and Tyson (1966) used the DAM test in order to investigate body representation in CP, finding frequent anthropometric deviations and lacking of body parts in a subset of drawings performed by hemiplegic children, probably reflecting children’s projection of their own specific physical impairment. However, these observations were not translated in quantitative terms, nor authors required systematically a self-portrait.

The view that the representation of the “self” in the generic DAM test is not firmly established (Harris, 1963) led some authors to prefer the self-portrait as an elective pictorial tool aimed to investigate children’s self-body representation. Indeed, Morin and coworkers have shown that the self-portrait may give access to imaginary and symbolic aspects of subjectivity in normal subjects (Morin and Bensalah, 1998), and to the subjective effects of alterations in body representation in patients with brain lesions (Morin, 1998; Morin et al., 2001). In this regard, Morin et al. (2003) collected 161 portraits performed by hemiplegic stroke patients. Interestingly, these authors reported in a subset of right brain injured patients a dissociation between self- and other-portraits: while drawing a “neglected” self-portrait, they spontaneously drew a complete image of others. These discrepancies persuaded the authors to embrace the idea that unilateral defects of portraits may selectively reflect the subjective alteration of the own body representation.

Asymmetrical self-portraits were not a constant feature in adult hemiplegic patients (Morin, 1993; Morin et al., 2003). This finding induced authors to support a brain-damage onset-dependent hypothesis, postulating that body representation (in particular its sensorimotor side, i.e., body schema) mostly forms in the early development (Lacan, 1966; Morin et al., 2003). Thus, the relative timing between the stroke onset and the development of body schema/image could be a key determinant for the presence of asymmetrical features in self-portraits. In this regard, an ideal model is represented by perinatal stroke survivors, whose injury certainly precedes the body schema/image instantiation. Within such population, it is possible to evaluate whether the motor impairment selectively impacts on self-body representation, rather than on body representation in general.

By enrolling a population of children suffering from UCP due to perinatal stroke showing isolated and unilateral motor deficit with prevalent upper limb involvement, we accounted for: (1) the influence of symbolic disturbances or neglect on self-portraying abilities, (2) the impact of motor impairment on the ability to perform a drawing, and (3) the “unawareness” of the impairment due to the hemiparesis onset posterior to body schema/image establishment processes. Using the test of the human figure, we asked children to draw a self-portrait, a portrait of a hemiparetic peer whom they joined in a child-to-child rehabilitation protocol, and a portrait of a healthy classmate. As controls, 18 age- and sex-matched typically developing children were asked to perform a self-portrait and a portrait of the best-classmate. We finally compared the drawings evaluating the asymmetry of representation of upper limbs, thus providing for the first time to our knowledge a quantitative index of self-portraits asymmetry.

In this study, we hypothesized that children with UCP present a larger asymmetry in self-portraits relative to other portraits, and also relative to self-portraits of typically developing children. In addition, the direct comparison between self-portraits and the hemiplegic peer-portraits should reveal whether this asymmetry is specific for self-representation, or vice versa whether it is associated to the “hemiplegic condition” representation.

## Materials and Methods

The study was approved by the Local Ethical Committee (Comitato Etico Area Vasta Emilia Nord) and was conducted according to the Helsinki Declaration. Subjects belonging to the clinical group were recruited in cooperation with “Fight The Stroke” association ^1^, in the framework of a broader clinical rehabilitative protocol involving children with cerebral palsy. The families of the controls were enrolled in the realm of another study conducted in our Center on primary school children. Written informed consent was obtained from parents of each child involved. Nineteen UCP children undergoing a child-to-child rehabilitative protocol (clinical group) and 18 typically developing children (control group) were enrolled in the study. The rehabilitative protocol in which children with UCP were involved was composed by 30 daily sessions based on child-to-child interaction, with each participant interacting with another hemiparetic child, performing specific hand exercises. The interacting couples of children remained the same throughout the whole program, thus facilitating a social relationship between them.

Inclusion criteria of the clinical group were: age between 5 and 10; confirmed diagnosis of UCP; evidence of ischemic mono-hemispheric damage at brain MRI; Upper limb Modified Ashworth Scale (MAS) sum score < 2; Total IQ ≥ 70. Exclusion criteria were: attentive or sensory impairments; seizures not controlled by therapy; previous orthopedic surgery or botulinum toxin A injection in the upper limb within 6 months prior to study entry. Eighteen age- and sex-matched typically developing children were selected as controls. Evaluation of UCP and controls was conducted during a single session, in a clinical setting, according to the following procedures.

During the clinical evaluation, the following data were collected in children with UCP: neurological complete examination (verifying also the absence of body representation disorders in body-part pointing and naming, awareness of spatial notions and left-right orientation), Global hand motor skills using Besta Scale Global Score [Besta GS Rosa-Rizzotto et al. (2014)], upper limb’s spasticity by means of Modified Ashworth Scale (MAS) (Bohannon and Smith, 1987), hand manipulative pattern classification (HC) according to Ferrari et al. (Bassi and Ferrari, 2016) and total Intelligence Quotient (IQ) by WISC-IV battery (Wechsler, 2012). Then, visuospatial constructional ability and visual memory were evaluated with Rey-Osterrieth Complex Figure Test (ROFC) (Shin et al., 2006) administered both in copy and early recall conditions (the latter performed 10′ after figure visualization).

All children were asked to seat comfortably on a height-adjusted chair placed in front of a table and were provided with a set of pencils and white sheets. Children with UCP were asked to perform 3 drawings in the following order: a self-portrait (SP), a portrait of the best classmate-friend (FP), and a portrait of the hemiparetic child who joint them in the child-to-child rehabilitation program (HP). Controls were asked to perform a self-portrait and a portrait of the best classmate. To ensure a spontaneous body representation, no specific indication was given to children.

From the initial set of drawings, 9 triads performed by UCP children and 2 dyads performed by controls were excluded due to the presence of non-anthropomorphic representations or non-measurable body parts. Drawings by 10 UCP children and 16 controls were finally considered for analyses. The length of each represented limb, measured as the inter-joint distance between the shoulder and the wrist, was measured. An asymmetry index (AI), consisting in the difference between the upper limbs length expressed as percentage of their average, was computed according to the following formula: AI = $\left|\frac{\mathrm{Left}-\mathrm{Right}}{\mathrm{Left}+\mathrm{Right}}\right|$ × 2 × 100. Giving an example: if we consider a portrait with a left and right arm length, respectively of 5 and 4 cm, the AI = | (5–4)/(5+4)| × 2 × 100 = 22.22%.

After verifying that the normality assumption was not met by AI data, a Kruskal Wallis H test was conducted in order to investigate between-groups differences in AI in portrait types. Within-group AI difference across portrait types has been investigated through a non-parametric repeated measures analysis of variance by ranks (Friedman test). *Post hoc* comparisons were conducted through non-parametric test (Wilcoxon), and effect size was computed by means of Eta squared and Kendall’s W parameters for between- and within- group analyses. Subsequently, we tested whether asymmetry was correlated to age and/or to clinical variables indexing motor and cognitive functioning. By means of Spearman (ranked) test, the correlation between the AI and Age, IQ, Besta GS and HC were tested. This set of regressors was chosen to test whether age, intelligence level or motor functioning could impact on the AI. Significance threshold was set at 5%.

## Results

The demographic data, clinical features and brain imaging findings of children with UCP are shown in Table 1. The mean age of the 10 analyzed subjects with UCP (7 males, 3 females) was 7.06 ± 1.90 years. Overall, they presented mild hemiparesis with a mild level of spasticity (total MAS = 1.95 ± 1.34), a prevalent upper limb involvement associated to a significant hand motor deficit (Besta GS = 0.48 ± 0.38). According to the HC, 2 subjects belonged to type I (“integrated hand”), 2 to type II (“semi-functional hand”), 3 to type III (“synergic hand”), 3 to type IV (“imprisoned hand”). Visuo-spatial abilities evaluated with ROCF test showed values within ± 2 z-score for both *copy* and *recall* conditions (mean z-score = −0.09, range [−2, +1.65], mean z-score = −0.44, range [−1.92, + 0.91], respectively), according to the Italian pediatric normative (Rey, 1968) (see Table 1 for individual ROCF z-scores collected in *copy* condition).

**Table 1:** Demographical data, clinical features and radiological findings of children with UCP.

ID	Sex	Age (y)	AH	Total IQ	ROCF (z-score)	Besta GS	SP-AI	HP-AI	FP-AI	MRI findings
1	M	7	R	103	n.a.	1.09	36%	20%	29%	Left parieto-occipital gliosis areas. Mild left ventriculum dilatation.
2	M	7	L	85	n.a.	0.05	107%	71%	13%	Right fronto-temporo-parietal gliosis in MCA territory. Severe right ventriculum dilatation. Corpus callosum hypotrophy.
3	M	8	L	103	1.65	0.73	10%	11%	7%	Right basal ganglia T1 hypointense areas. Mild right ventriculum dilatation.
4	F	10	R	70	−2	0.16	45%	34%	25%	Left fronto-temporo-insulo-parietal malacic areas. Severe right ventriculum dilatation. Corpus callosum hypotrophy.
5	M	7	R	80	−0.2	0.22	67%	4%	15%	Left fronto-temporo-parietal malacic areas in MCA territory. Left ventriculum dilatation.
6	M	8	R	80	−1.85	0.84	19%	15%	6%	Severe dilation of frontal horn of right ventriculum.
7	F	8	L	107	0.15	0.10	15%	13%	11%	Right fronto-temporo-insulo-parietal malacic areas in MCA territory. Mild right ventriculum dilatation.
8	F	5	R	95	1.5	0.25	20%	21%	4%	Left basal ganglia T1 hypointense areas. Severe Left ventriculum dilatation
9	M	5	R	100	−1	0.88	45%	8%	10%	n.a.
10	M	5	L	105	1	0.48	26%	20%	17%	Right periventricular gliotic area. Mild right ventriculum dilatation.

*M, male; F, female; AH, affected hand; ROCF, Rey-Ostereith Complex Figure Test; Besta GS, Besta global score; ΔBestaGS to peer, difference in Besta Global Score relative to peer (positive values indicate better hand functioning relative to peer); SP-AI, self portrait asymmetry index; HP-AI, hemiparetic portrait asymmetry index; FP-AI, healthy classmate portrait asymmetry index; n.a., not available. Although not part of the analysis, MRI findings have been added in the table in order to demonstrate the presence of a predominantly unilateral brain lesion in the enrolled subjects, as well as to enrich the clinical data of our sample.*

**FIGURE 1:**
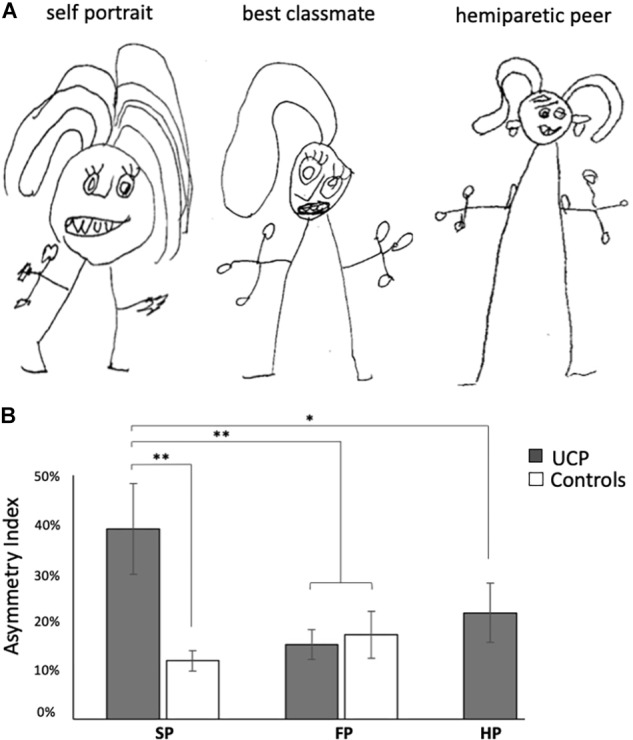
**(A)** Example of portraits performed by a child: self-portrait, portrait of the hemiparetic peer with similar clinical conditions (5 years-old, unilateral cerebral palsy with prominent upper-limb motor impairment), portrait of best classmate. Note – only in self-portrait – the asymmetrical representation of upper-limb, with the paretic hand smaller than the contralateral one and without fingers. **(B)** Asymmetry index differences across different portrait types in children with UCP and controls. SP, self portrait; FP, portrait of the best classmate-friend; HP, portrait of the hemiparetic peer. Bars indicate SEM; ^∗^*p* < 0.05, ^∗∗^*p* < 0.01.

Neurological examinations show neither neglect nor hemiasomatognosia. All children were able to name their body parts correctly, no orientation abnormalities were detected, and spatial concepts were preserved. No children were excluded due their clinical profile. Overall, drawing were highly heterogeneous in terms of graphic style, with the precision and richness of details varying according to the age. However, an internal consistency was evident within-subject, with the three drawings presenting recurrent elements and a common graphical style (see Figure 1A).

The control group was composed by 16 typically developing children (10 M, mean age 7.37 ± 1.75). As expected, ROCF test performed in controls returned normal values for both *copy* and *recall* conditions (mean z-score = 1.51, range [−0.5, 2.5] and mean z-score = 0.92, range [−0.86, 1.85], respectively).

The Kruskal-Wallis H test showed a statistically significant difference in AI in SP between two groups [χ^2^(1) = 11.025, *p* = 0.001, effect size: η^2^ = 0.418]. *Post hoc* contrasts indicated a significantly greater AI in self-portraits by UCP children relative to Controls (*p* < 0.001, see Figure 1B).

Within UCP group, the Friedman test applied to the AI rendered a chi-square value of 11.4, returning a significant effect of portrait type (*p* = 0.003, effect size: Kendall’s *W* = 0.57). In particular, UCP children represented upper limbs more asymmetrically in self-portraits relative to other drawings (mean AI for SP: 39%, FP: 14%, HP: 22%). *Post hoc* contrasts indicated a significantly greater AI in self-portraits in comparison both to FP (*p* = 0.005) and HP (*p* = 0.013) (see Figure 1B). Moving to control group, no AI significant difference between SP and FP was found.

The study of clinical-demographical regressors on AI of self-portraits did not show any significant correlation. Besides, differential regressors related to the hemiparetic peer did not show significant correlations with the difference between SP and HP asymmetry indexes.

## Discussion

The aim of the present study was to evaluate self-body representation in hemiparetic children affected by UCP with predominant upper limb involvement and to compare this pictorial representation to portraits of both hemiparetic and healthy peers. For this purpose, we evaluated the upper limb asymmetry in the three portrait types, which resulted significantly higher in self-portraits compared to both hemiparetic and healthy peers ones. Of note, self-portraits produced by typically developing children showed no significant difference in asymmetry, neither in comparison to portraits of others performed by the same group, nor relative to the portraits of others performed by children with UCP. This finding led us to regard the asymmetry of upper limbs in self-portraits as a specific signature of hemiparetic children.

The detection of asymmetries in own upper-limb representation in children with UCP is coherent with a previous work conducted by Abercrombie and Tyson (1966) on children suffering from cerebral palsy, in which the occurrence of unbalanced representations of upper limbs were reported in children with an unilateral brain damage. However, these authors used the Draw-a-Man test (Goodenough, 1975) as a projective test, implicitly making children represent their own body image. Differently from these authors, we explicitly asked children to produce both self- and classmate- portraits. The possibility to directly compare these drawings allowed us to verify whether upper limb asymmetry reflects an alteration of the own body image rather than a deviant representation of human body in general. Two are the major strengths of this approach. On one side, the within-subject comparison allowed us to rule out the contribution of subject peculiarities in drawing. On the other side, despite diagnosed for UCP, our clinical sample was free from visuospatial and symbolic disturbances, hemiasomatognosia and neglect, thus controlled for major disorders affecting pictorial representation.

The finding of a three-times higher level of asymmetry in self vs. classmate representation is in line with a previous work of Morin et al. (2003). These authors conducted a multivariate analysis evaluating 161 portraits performed by adult stroke patients (including both self-portraits and portraits of others). As expected, authors reported frequent “unilateral lacks” in right brain injured patients’ drawings, attributing these difficulties to several aspects of hemineglect. However, some right-hemiparetic patients, despite drawing a “neglected” self-portrait, spontaneously drew a complete image of others, leading to postulate that unilateral defects of portraits may selectively reflect an alteration of body self-representation.

Although in line with our findings, whether this deviant representation constitutes a signature of the *self*-representation, or rather it is a more general representation of the *hemiparetic condition*, is still unclear. To address this issue, we required participants to portray also a hemiparetic peer with whom they had been experiencing a daily interaction in the previous month. This condition allow us to demonstrate that the asymmetrical picturing of upper limbs constituted a signature of the *self*-representation, favoring the view that self-portrait features are grounded in a first-person, sensorimotor bodily experience.

No correlation was found between the asymmetry in upper limb representation and indices of motor functioning. However, the small sample size and the heterogeneity of the investigated population in terms of brain lesions require further studies to reveal a possible link between these two domains.

## Conclusion

In conclusion, our data indicate that UCP with predominant upper limb deficit affects body self-representation, but not body-representation in general. We suggest that the upper limb asymmetry does not constitute a picturing of pathological condition, but rather it may reflect the experienced status of motor functioning, that is valid only for one’s own. We propose that evaluating self-portrait in hemiparetic children undergoing pediatric neurorehabilitation programs and quantifying the asymmetry of the self-representation could provide a valuable index of self-perceived functioning. Such procedure, well-suited for pediatric age, would enrich the clinical picture of the patient by adding a psychometric information to clinical outcomes, enabling clinicians to collect information not easily obtainable in pediatric patients.

## Author Contributions

AN collected the data and drafted the manuscript. PP and PA revised the manuscript. MF-D interpreted the data and revised the manuscript.

## Conflict of Interest Statement

The authors declare that the research was conducted in the absence of any commercial or financial relationships that could be construed as a potential conflict of interest.

## Footnotes

1 www.fightthestroke.org

## References

[B1] Abercrombie M. L. Tyson M. C. (1966). Body image and draw-a-man test in cerebral palsy. *Dev. Med. Child Neurol.* 8 9–15. 10.1111/j.1469-8749.1966.tb08267.x 5331749

[B2] Bassi B. Ferrari A. (2016). *L’arto Superiore Nella Paralisi Cerebrale Infantile: Aspetti Clinici e Possibilità Terapeutiche.* Padova: Piccin.

[B3] Bohannon R. W. Smith M. B. (1987). Interrater reliability of a modified Ashworth scale of muscle spasticity. *Phys. Ther.* 67 206–207. 10.1093/ptj/67.2.206 3809245

[B4] Cooke E. (1885). Art teaching and child nature. *London J. Educ.* 7 462–465.

[B5] de Vignemont F. (2010). Body schema and body image–pros and cons. *Neuropsychologia* 48 669–680. 10.1016/j.neuropsychologia.2009.09.022 19786038

[B6] Gallagher S. (2005). *How the Body Shapes the Mind.* Oxford: Oxford University Press 10.1093/0199271941.001.0001

[B7] Goodenough F. L. (1975). *Measurement of Intelligence by Drawings.* New York, NY: Arno Press.

[B8] Harris D. B. (1963). *Children’s Drawings as Measures of Intellectual Maturity.* New York, NY: Harcourt, Brace and World Inc, 46–48.

[B9] Lacan J. (1966). *The Mirror Stage as Formative of the I Function as Revealed in Psychoanalytic Experience.* New York, NY: Norton & Company.

[B10] Lampe R. Lützow I. Blumenstein T. Turova V. Alves-Pinto A. (2016). Critical analysis of children’s drawings as a diagnostic tool for body schema and body image disorder in cerebral palsy. *Neurosci. Med.* 07 133–148. 10.4236/nm.2016.74014

[B11] Machover K. (1949). *Personality Projection in the Drawing of Human Figure: A Method of Personality Investigation.* Springfield, IL: Charles C. Thomas 10.1037/11147-000

[B12] Matthews J. (2003). *Drawing and Painting: Children and Visual Representation.* Available at: http://sk.sagepub.com/books/drawing-and-painting-2e

[B13] Morin C. (1993). Autoportraits de patients après AVC. *La Psychanalyse de l’Enfant* 13 140–157.

[B14] Morin C. (1998). Corps, image, objet en neurologie. *Bulletin de l’Association Freudienne Internationale* 77 7–11.

[B15] Morin C. Bensalah Y. (1998). The self-portrait in adulthood and aging. *Int. J. Aging Hum. Dev.* 46 45–70. 10.2190/U3P8-8YBF-0DL0-HV2P 9534075

[B16] Morin C. Pradat-Diehl P. Robain G. Bensalah Y. Perrigot M. (2003). Stroke hemiplegia and specular image: lessons from self-portraits. *Int. J. Aging Hum. Dev.* 56 1–41. 10.2190/F0G2-GW5C-4WG0-KBWL 12940448

[B17] Morin C. Thibierge S. Perrigot M. (2001). Brain, body image and language: a psychoanalytic perspective. *J. Mind Behav.* 22 69–89.

[B18] Rey A. (1968). *Reattivo Della Figura Complessa - Manuale.* Firenze: Organizzazioni Speciali.

[B19] Rosa-Rizzotto M. Visonà Dalla Pozza L. Corlatti A. Luparia A. Marchi A. Molteni F. (2014). A new scale for the assessment of performance and capacity of hand function in children with hemiplegic cerebral palsy: reliability and validity studies. *Eur. J. Phys. Rehabil. Med.* 50 543–556. 24732444

[B20] Shin M.-S. Park S.-Y. Park S.-R. Seol S.-H. Kwon J. S. (2006). Clinical and empirical applications of the rey–osterrieth complex figure test. *Nat. Protoc.* 1 892–899. 10.1038/nprot.2006.115 17406322

[B21] Wechsler D. (2012). *WISC-IV Wechsler Intelligence Scale for Children Quarta Edizione. [Manuale di Somministrazione e Scoring].* Firenze: Giunti OS.

[B22] Wittmann B. Barber C. A. (2013). “Neolithic childhood: children’s drawings as prehistoric sources,” in *Res: Anthropology and Aesthetics*, ed. Pellizzi F. (Chicago, IL: The University of Chicago Press), 125–142. 10.1086/690982

